# Antimicrobial effects of pulsed light activated TiO_2_-Polylactic acid film

**DOI:** 10.1016/j.heliyon.2024.e38891

**Published:** 2024-10-02

**Authors:** Tony Z. Jin, Xuetong Fan, Sudarsan Mukhopadhyay

**Affiliations:** Eastern Regional Research Center, Agricultural Research Service, U.S. Department of Agriculture, 600 East Mermaid Lane, Wyndmoor, PA, 19038, USA

**Keywords:** Titanium dioxide, Polylactic acid, Pulsed light, Antimicrobial, Biodegradable, Food packaging

## Abstract

A study was conducted to evaluate the antimicrobial effect of TiO_2_ incorporated polylactic acid (PLA) films, activated by pulsed light (PL). TiO_2_-PLA films with 0, 0.5, 5, 10, and 20 % TiO_2_/PLA ratio were developed by the solvent casting method. Populations of *Escherichia coli* and *Listeria* on film surfaces were determined after the films were PL-treated in three ways: PL treatment on the inoculated film surface; PL treatment from the reverse side of the inoculated film surface; PL treatment before the inoculation on the film surface. A 5 s PL treatment reduced *E. coli* populations on PLA film from 6.2 to 2.5 log CFU/cm^2^ and on TiO_2_-PLA film from 6.2 to 1.1 log CFU/cm^2^, indicating the combined antibacterial effect of PL and TiO_2._ Light-activated TiO_2_-PLA films maintained antibacterial activity against *E. coli* and *L. innocua* for 2 h after the 5 s PL treatment, as the activated TiO_2_-PLA films were still able to reduce bacterial populations by up to 2.5 log CFU/cm^2^. These findings suggest that pre-activated TiO_2_-PLA films have potential for packaging foods that are sensitive to UV light.

## Highlights

• A composite packaging film with TiO_2_-Polylactic acid (PLA) was developed.
• Antibacterial activity of TiO_2_-PLA film was activated by pulsed UV light (PL).
• TiO_2_ in PLA film significantly enhanced antibacterial effect of PL treatment.
• TiO_2_-PLA film maintained the antibacterial activity after PL-activation.
• TiO_2_-PLA film has potential application in antimicrobial food packaging.

## 1 Introduction

Foods can be protected from physical damage through proper food packaging. However, conventional food packaging materials typically do not possess antimicrobial properties and may even be contaminated with pathogenic and spoilage microorganisms during manufacturing, use and/or handling [1]. This could cause cross-contamination of food items, reduce their shelf life, and increase health risks [2,3].

The strong oxidative and reductive power of titanium dioxide (TiO_2_) was discovered and described as the Honda-Fujishima effect in 1972 [4]. Since then, titanium dioxide compounds have been incorporated into popularly recognized photocatalyst materials for bacterial inactivation, which can be used even under conditions where available UV light is limited. Li et al. [5] reported that modified TiO_2_ powders could absorb visible light radiation, and thus induce antibacterial activity. Other uses of these materials include self-cleaning surface coatings, which can be applied for use in places such as medical centers [6,7]. TiO_2_ nanoparticles are considered a benchmark photocatalyst, as well as an antibacterial agent, due to their large surface area, high chemical stability, high catalytic activity, and low cost [8]. Using the antimicrobial properties of TiO_2_, various biopolymer-based antimicrobial food packaging films have been developed [[9], [10], [11], [12], [13], [14]].

Pulsed light (PL) utilizes short, intense pulses of broad-spectrum light ranging from UV to near infrared (200–1100 nm) to inactivate microorganisms. The antimicrobial efficiency of PL is higher than that of continuous-wave low-pressure UV irradiation (CW-UV) [15]. This is due to the high-peak power of PL, along with its ability to deliver stored energy over short durations, typically 1 to 10 pulses per second. PL exhibits its antimicrobial activity through photochemical and photothermal effects [15]. PL treatments for inactivating *Salmonella* on cherry tomatoes, and *E. coli* O157:H7 on romaine lettuce have been investigated [16,17]. However, there is very limited information available regarding the use of PL for the activation of TiO_2_.

Antimicrobial efficacy is an essential parameter for antimicrobial food packaging that inactivates spoilage and foodborne pathogens to extend the shelf life and enhance the safety of packaged foods. Recently, many studies have focused on developing environmentally friendly and biodegradable food packaging materials. Several studies have demonstrated that TiO_2_ can be incorporated into biopolymer-based packaging materials. The U.S. Food and Drug Administration (FDA) has approved the use of inorganic TiO_2_ nanoparticles as a safe compound, to be used in food and food contact materials at restricted quantities below 1 % of the food weight [18], which opens a door for its application in food and food packaging. Chitosan-TiO_2_ nanocomposites were proven to possess favorable mechanical, thermal, and photocatalytic properties [19,20] and have been shown to have potential for application as an antibacterial fabric [21], an antibacterial coating [22], as well as in various fields, including food preservation [23] and controlled drug-delivery systems [24]. However, the hydrophilic nature and weak mechanical properties of chitosan limit its application in food packaging. Other biopolymers, such as starch [12,14], and gellan gum [11] have been incorporated into biodegradable food packaging materials, with similar problems. The polylactic acid (PLA) polymer is one of several biodegradable food packaging materials derived from renewable resources such as corn, wood residues, or other biomass. The physical and mechanical properties of PLA are comparable to those of many petroleum-based polymers and are superior to those of chitosan or other biopolymers. Several studies have demonstrated that PLA can be used as an excellent antimicrobial carrier incorporated into antimicrobial food packaging [[25], [26], [27], [28], [29], [30], [31], [32]]. Asadi and Pirsa [33] developed a new film based on polylactic acid (PLA) with TiO_2_ and lycopene. The authors reported that the addition of TiO_2_ not only increased the antibacterial effect, but also significantly improved the physical and chemical properties of the film. However, there has not been a reported study combining pulse light treatment on PLA films with TiO_2,_ neither on the different PL activation methods.

As mentioned above, the antibacterial activity of films composed of TiO_2_ and PLA have been studied and reported. However, there was very limited information available about the antibacterial activity of TiO_2_-PLA films which is activated directly and indirectly by pulse UV light. Specifically, there were no reports on the pre-activated TiO_2_-PLA films by pulsed light, that is, if PL could pre-activate TiO_2_ film before food packaging, which would have significant advantages for foods with ingredients that are sensitive to direct UV light treatment. Therefore, in the present study, we compared directly and indirectly PL treatments on TiO_2_-PLA films and assessed the antimicrobial properties of developed PLA-TiO_2_ films with various TiO_2_ concentrations against *E. coli* and *Listeria*. The gained knowledge and the best method from this study could be used to further develop antimicrobial food packaging.

## 2 Materials and methods

### 2.1 Materials

Titanium oxide nanopowder (21 nm primary particles size, range from 1 to 150 nm; >99.5 %) was purchased from Sigma-Aldrich-Fluka (St Louis, MO, USA). Methylene chloride and glass Petri dishes (100 × 15 mm) were sourced from Fisher Scientific (Fairlawn, N.J., U.S.A.). PLA resin was obtained from Natureworks (Minnetonka, Minn., U.S.A.). Tryptic soy broth (TSB), Palcam agar, and tryptic soy agar (TSA) were purchased from BBL/Difco Laboratories (Sparks, MD, USA). Palcam selective supplement was from Oxoid (Basingstoke Hampshire, England).

### 2.2 Preparation of TiO_2_-PLA film

**Fig. 1:**
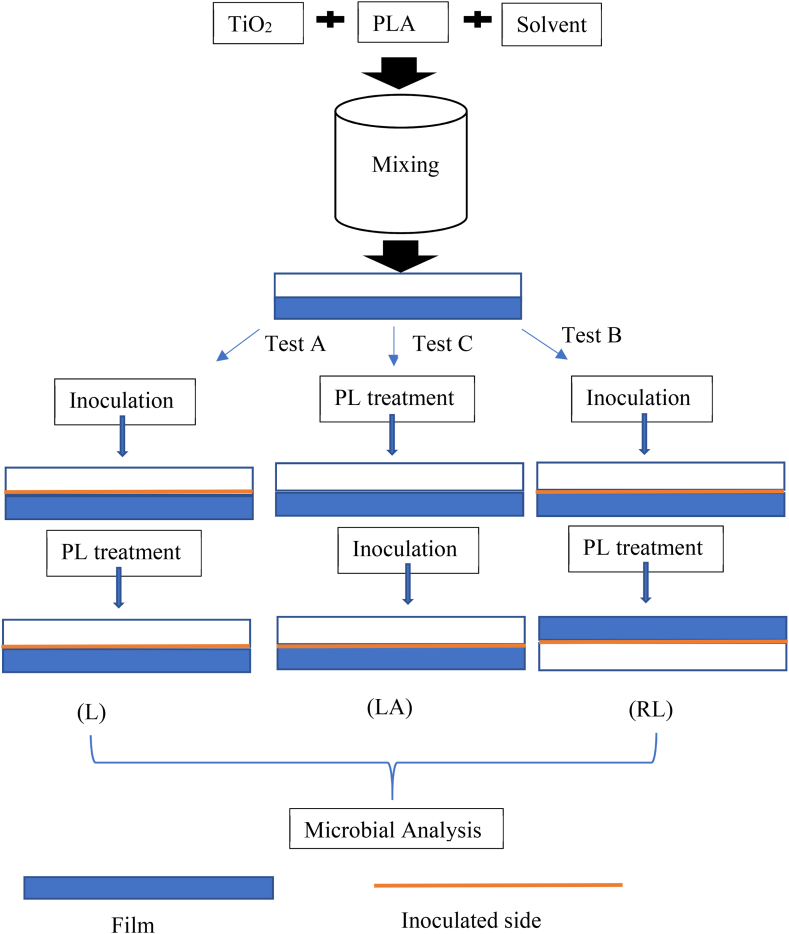
Procedures for film-making, inoculation and PL treatments.

The solvent casting method was used for generating TiO_2_-PLA films (Fig. 1). One gram of PLA resin powder, in addition to designated concentrations of TiO_2_ nano powder (described below), were mixed into 10 mL methylene chloride. The mixture was stirred using a magnetic stir bar for 24 h to distribute TiO_2_ evenly into the PLA polymer. The ratios of TiO_2_/PLA were 0, 0.5, 5, 10, and 20 % wt, and were labeled as P0, P005, P05, P1, and P2, respectively. Ten mL of each TiO_2_/PLA solution was distributed to an individual glass Petri dish and each Petri dish was air-dried at room temperature (21 °C) to allow for solvent evaporation. Petri dishes were then stored in the absence of light until they were ready for experimental use. Films were peeled from the Petri dishes before tests. The average thickness of films was 0.15 mm.

### 2.3 Color and light transmittance

Color (whiteness, *L* value) of each film was measured by a portable colorimeter (CM-700 d, Konica Minolta, NJ, USA). *L* value included ranges from 0 (blackness) to 100 (whiteness).

The ultraviolet (UV) and visible light barrier properties of the films were measured at selected wavelengths, from 200 to 800 nm, using a UV spectrophotometer (Genesys 10UV, Thermo Electron Corp., Madison, MI, USA) [11].

### 2.4 Pulsed light treatments

A laboratory scale PL unit (Steripulse-XL RS-3000, Xenon Corp., Wilmington, MA) was used for PL treatments. The unit was equipped with a treatment chamber, a controller module, and an air-cooling module, which generated a pulse with a 360 μs width and 3 pulses per second with width of 500 μs each pulse and wavelength 180–1000 nm of which 40 % belongs to the UV region. The distance between the lamp and the quartz window in the PL unit was 5.8 cm, and the distance between the bottom of the treatment chamber and the quartz window was 16 cm. The energy delivered to the film surface at this distance was 0.35 J/cm^2^/pulse as measured by Vega laser power meter (OPHIR Photonics, North Logan, UT, USA) [16,17].

### 2.5 PL treatment of films

The inoculated films (prepared in section 2.6) were dried for 1 h under the biohood to allow for bacterial attachment, prior to PL treatments.

The antimicrobial activity of the films against *L. innocua* and *E. coli,* inoculated onto the film surface, were investigated in three different ways (Fig. 1).

A. Film inoculation followed by PL treatment on the inoculated film surface (L). The film sample was inoculated and dried for 1 h, after which it was subject to PL treatment.
B. Film inoculation followed by PL treatment from the reverse side (RL). The film sample was inoculated and dried for 1 h, after which it was turned over and subject to PL treatment on the reverse side.
C. Film PL treatment followed by inoculation (LA). The film sample was subjected to the PL treatment and then inoculated with the respective bacterial suspension on the same side within 1 h.

### 2.6 Determination of antimicrobial properties of films activated by PL

#### 2.6.1 Inoculum preparation

*E. coli* ATCC 23716 (K12) and *Listeria innocua* ATCC 51742 were used in this study. They were obtained from the American Type Culture Collection (Manassas, VA., USA.). Frozen stock cultures of each strain were cultured independently in 30 mL Tryptic Soy Broth in sterile 50 mL conical tubes at 37 °C for 18 h. The overnight pure broth cultures (in 10 mL Tryptic Soy Broth) were washed by centrifugation at 3500 rpm for 10 min at 4 °C. Each culture was then resuspended in 10 mL of autoclaved 0.1 % peptone water [30].

#### 2.6.2 Microbial inoculation

A method described by Zhang et al. [34] was used with some modification. Five hundred μl of *E. coli* and *L. innocua* inoculum, respectively, prepared in above, were evenly distributed on the surface of each film (∼78 cm^2^) inside each glass Petri dish using a sterile L-shape spreader (Sigma-Aldrich-Fluka, St Louis, MO, USA). The initial bacterial load on each film was approximately 10^5^ cells/cm^2^. The inoculated films were dried for 1 h under the biohood to allow for bacterial attachment, prior to PL treatments as described in Section 2.5.

#### 2.6.3 Microbial analysis

Antimicrobial properties were determined using a liquid incubation method as described by Jin & Zhang [29]. Following the PL film treatment, each film sample was assessed to determine the survival of *E. coli* or *Listeria* populations on each surface. One mL of sterile peptone water (0.1 %) was added to the Petri dish. The film surface was then swabbed with sterile cotton tips. A 100 μL specimen was aspirated from each Petri dish. Serial dilutions of the resultant bacterial suspensions were prepared in 0.1 % peptone water, and 100 μL of the appropriate dilutions were surface plated onto Palcam agar plates with Palcam selective supplement to determine *Listeria* populations, or onto TSA plates to determine *E. coli* K12 populations. After incubating the plates for 24–48 h at 37 °C, colony forming units (CFU) on each plate were counted and recorded for data analysis.

### 2.7 Statistical analysis

All experiments were conducted independently in triplicate with three film samples (Petri dishes) per light treatment. Raw bacterial populations from each experimental trial were transformed to log_10_ values, and means were determined. Data were analyzed by SAS GLM (General Linear Model) procedure, and significant differences (*p* < 0.05) in the reduction of bacterial populations between treatments were determined by Duncan's multiple range test via SAS version 9.4 (SAS Institute, Inc., Cary, NC).

## 3 Results

### 3.1 Appearance, color, and light transmission of PLA films with TiO_2_ powder

**Fig. 2:**
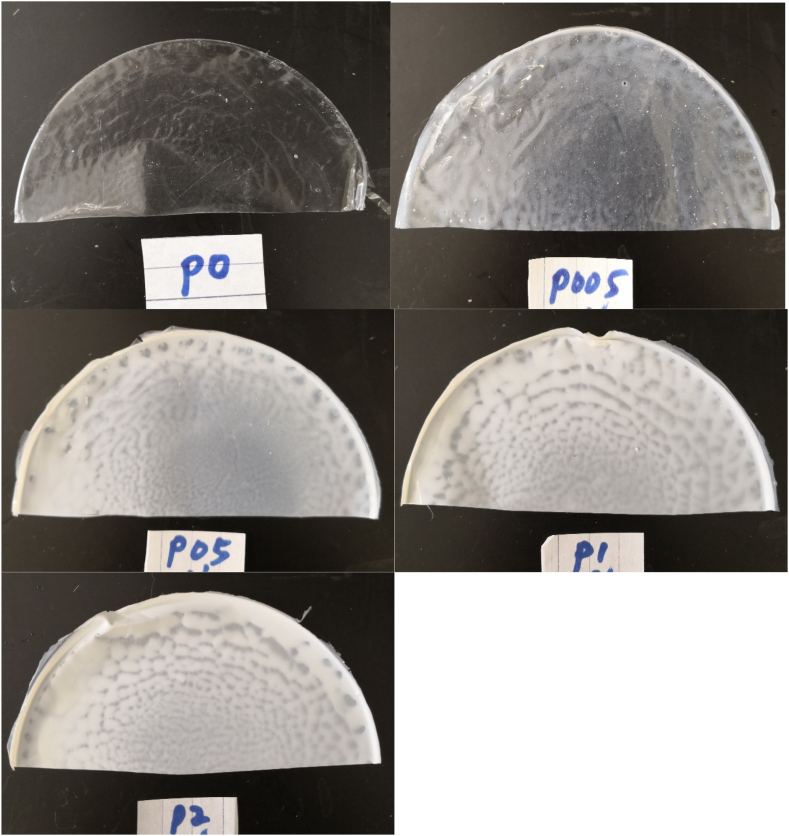
Appearance of films. The ratios of TiO_2_/PLA were 0 (P0), 0.5 % (w/w) (P005), 5 % (w/w) (P05), 10 % (P1) (w/w), 20 % (w/w) (P2).

The images in Fig. 2 reveal the physical appearance of PLA films with TiO_2_ concentrations ranging from 0 to 20 % (P0 to P2). The images reveal that the optical transparency decreased, with increasing concentrations of TiO_2_. Correspondingly, film whiteness increased commensurate with increasing TiO_2_, as revealed in Table 1.

**Table 1:** Color (whiteness, *L*) and light transmission (*T*) of films.

Film	L				*T* (%)			
		200 nm	280 nm	350 nm	400 nm	500 nm	600 nm	800 nm
P0	37.1	86.3	83.5	85.0	87.4	88.8	89.5	89.9
P005	43.5	27.2	16.4	14.2	24.4	37.8	47.6	59.1
P05	67.4	5.5	2.6	0.5	2.7	5.85	9.7	17.6
P1	79.4	2.1	0.8	<0.1	1.1	1.8	2.6	5.1
P2	85.2	0.5	0.3	<0.1	0.1	0.7	1.1	1.4

Table 1 also lists the light transmission of films from 200 nm to 800 nm wavelengths. Light transmission (*T*) was influenced by TiO_2_ concentrations in the films; the increase of the concentration of TiO_2_ resulted in the decrease of *T*, from all the wavelengths from 200 to 800 nm. Chen et al. [11] also observed transmittance at 300–400 nm was lower than 5 % and the transmittance in the visible region (400–800 nm) was lower than 20 % when TiO_2_ particles were added to cellulose composite films. In our case, increase of TiO_2_ to 0.5 % (w/w) (P005) and above resulted in the transmittance falling below 20 % from 200 to 800 nm.

### 3.2 Antimicrobial effect of films by pulsed light activation

**Fig. 3:**
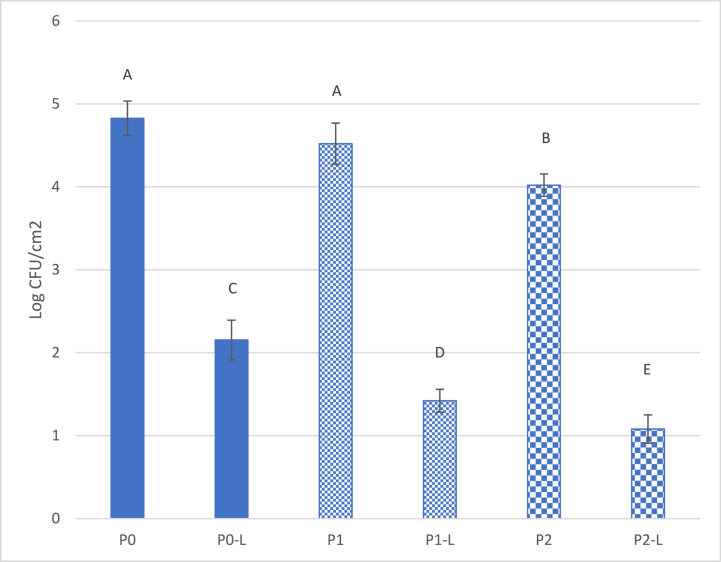
Survival of *E. coli* on films. P0, P1 and P2: No PL treatment. P0-L, P1-L, and P2-L: following 5 s PL treatment. Vertical bars indicate standard deviation and means with same letters are not significantly different (*p > 0.05*).

Results displayed in Fig. 3 indicate that pulsed light treatments also significantly (*p* < 0.05) influenced the antimicrobial effect of the films, regardless of the amount of TiO_2_ present.

The 5 s PL treatment, alone, reduced *E. coli* populations on films without TiO_2_ (P0 vs. P0-L) by 2.6 log CFU/cm^2^ and further reduced *E. coli* populations by 3.5–3.7 log CFU/cm^2^ on films with TiO_2_ (P1-L and P2-L). The bacterial counts on P1-L and P2-L films were 1.4 and 1.2 log CFU/cm^2^, respectively. These counts are significantly lower than those obtained on the P0-L film (2.15 log CFU/cm^2^), suggesting that TiO_2_ played an important role in effecting antimicrobial activity.

Additionally, an increase in the concentration of TiO_2_ in films from 10 % (P1) to 20 % (P2) increased the antimicrobial effect of both PL treated or non-PL treated films (Fig. 3); hence, P2 films were utilized in further studies for PL treatments.

### 3.3 Antimicrobial effect of films by PL treatment from the reverse side

**Fig. 4:**
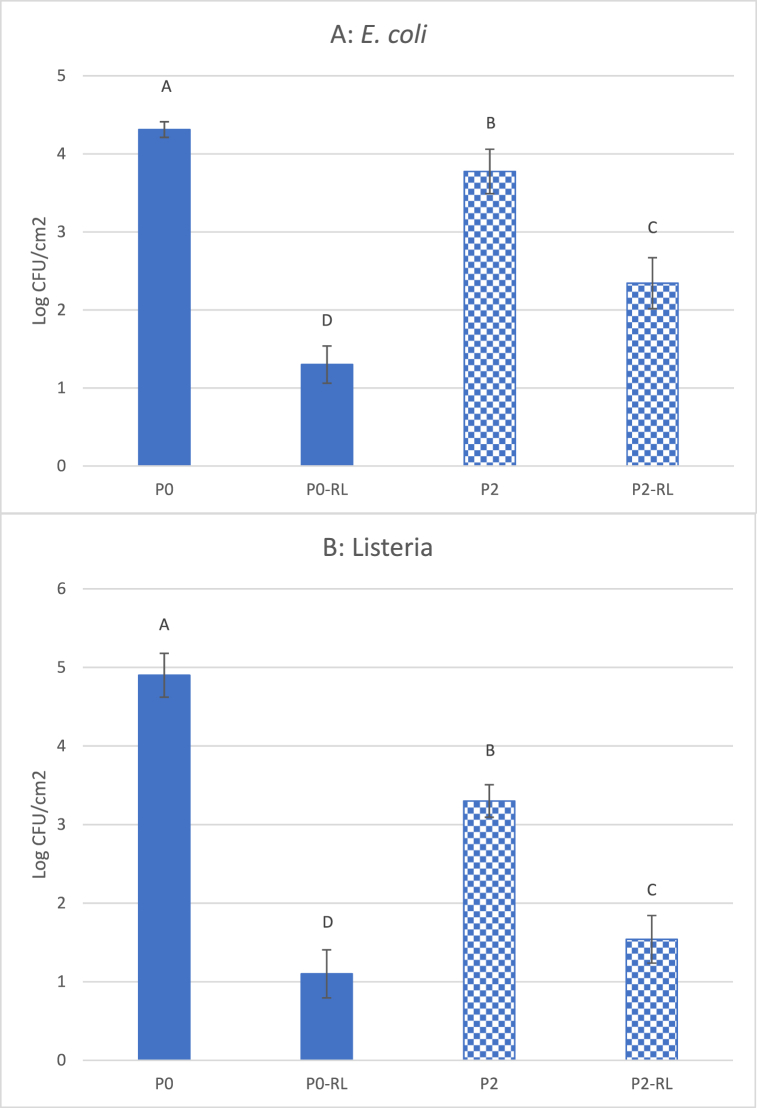
Survival of *E. coli* (A) and *Listeria* (B) on films following 5 s reversed PL treatment. Vertical bars indicate standard deviation and means with same letters are not significantly different (*p > 0.05*).

The purpose of film inoculation, followed by PL treatment on the reverse side (RL), was to investigate the influence of the opacity of the film on activating antimicrobial activity. Fig. 2 and Table 1 reveal that the translucency of films decreases with increasing concentrations of TiO_2,_ which could influence the effect of light treatments. In this study, the influence of film translucency/opacity was investigated when the inoculated films were subject to PL treatment from the reverse side. As expected, both *E. coli* (Fig. 4A) and *Listeria* (Fig. 4B) survived in greater numbers when exposed to films without TiO_2_ (P0) in comparison to those with TiO_2_ (P2). This indicates that opaque films block UV penetration and reduce their antibacterial effect. However, 5 s of PL treatment with P2 film still reduced populations of both *E. coli* and *Listeria* by > 2 log CFU/cm^2^. Obviously, these reductions were attributed to the antimicrobial properties of TiO_2_ alone. Chi et al. [35], who wrapped mangoes with PLA-TiO_2_ films and stored for 15 days without light treatment, found that the total bacterial count of mangoes, packaged with TiO_2_ -PLA films, was 2 log CFU/mL lower than samples packaged with other films, following 3 days of storage.

### 3.4 Antimicrobial effects of PL pre-activated films

**Fig. 5:**
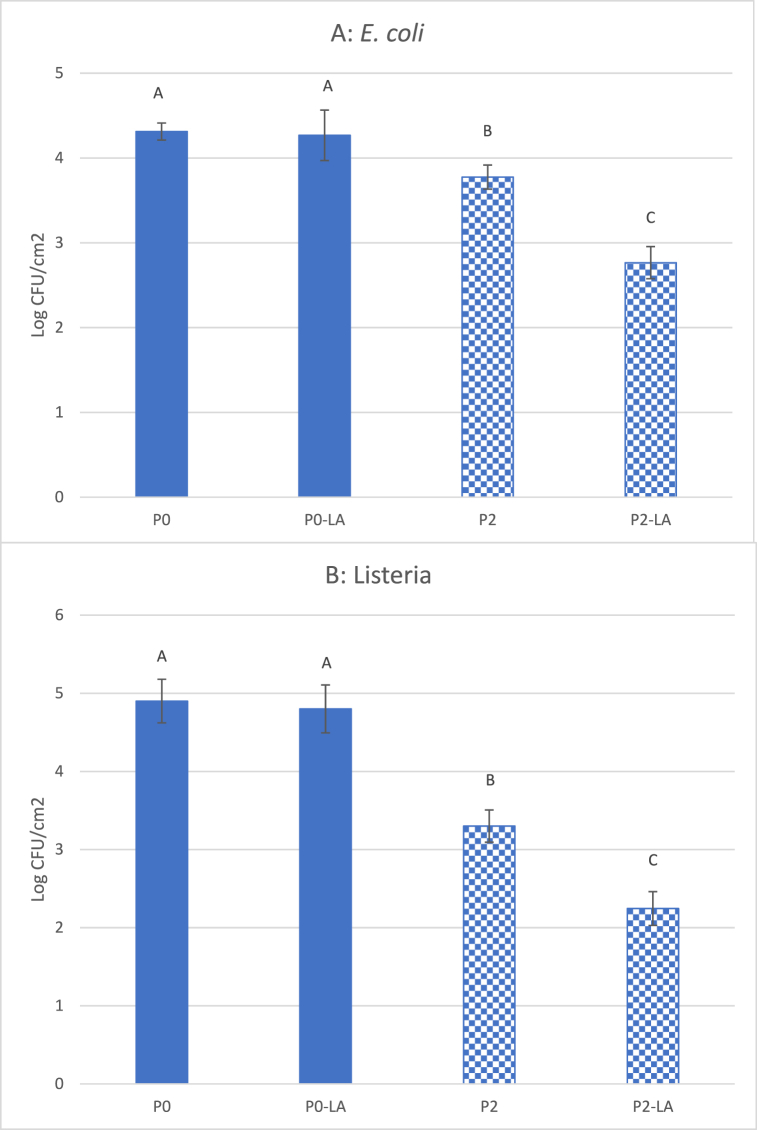
Survival of *E. coli* (A) and *Listeria* (B) on pre-PL treated films. Vertical bars indicate standard deviation and means with same letters are not significantly different (*p > 0.05*).

P2 films were subjected to treatment with PL for 5 s prior to inoculating with either *E. coli* or *Listeria*. Bacterial populations were assessed 2 h after inoculation. As expected, the results displayed in Fig. 5 indicate that pre-PL treatment did not significantly contribute to any reduction in microbial populations of *E. coli* (A) or *Listeria* (B) on films without TiO_2_ (P0-LA). However, P2 films activated by PL (P2-LA), prior to inoculation, still retained strong antibacterial activity. These films reduced populations of both *E. coli* and *Listeria* by 1.5–2.6 log CFU/cm^2^, when compared with those on P0 or P0-LA films (Fig. 5). This may be the first report of antimicrobial pre-activated TiO_2_ packaging materials, as there is currently no information available regarding PL pre-activated packaging materials or its application.

It is also observed that the TiO_2_-PLA film showed more antibacterial effects against *Listeria* (Gram +) than *E. coli* (Gram -) (Fig. 4, Fig. 5), which agrees with previous reports [13,36]. This could be due to the cell membrane structure, as Gram-negative bacteria (*E. coli*) have triple-layered cell wall structure (the cytoplasmic inner membrane, the thin peptidoglycan middle layer, and the outer membrane) that could prevent many molecules from crossing the cell membrane [37], while the Gram + *Listeria,* known to have a very thick cell wall, may be more susceptible to the treatment.

## 4 Discussion

This study demonstrates the antimicrobial properties of TiO_2_-containing films. The biocidal effect of TiO_2_ films activated with light treatments consists of three parts: 1. Effect of light; 2. Effect of TiO_2_; 3. The combined effect of TiO_2_ and light.

The biocidal effect of continuous UV or pulsed light on foods or packaging materials is well known. Results displayed in Fig. 3 indicate that PL treatments significantly reduced bacterial populations on films with or without TiO_2_. Pulsed light utilizes short, intense pulses of broad-spectrum light; hence, the antimicrobial efficiency of PL is higher than that of continuous-wave UV irradiation. The antimicrobial activity of PL is due to the interaction between the photochemical and photothermal effects [15]. The biocidal effect of PL treatment alone can be seen from the P0-L results, while additional TiO_2_ particles in films (P1 and P2) significantly enhanced the reduction of bacterial populations, inducing the combined biocidal effects (Fig. 3).

The antimicrobial function of the TiO_2_-added film is due to the excellent photocatalytic activity of TiO_2_ [37]. The photoactivation of TiO_2_ is initiated by adsorption of a photon with energy equal to or greater than the band gap of TiO_2_ [38]. The charged TiO_2_ particles can react with either electron acceptors or electron donors adsorbed on the surfaces, such as O_2_, to generate free hydroxyl radicals, superoxide radicals, or peroxide, which contribute to the destruction of bacterial cells [[39], [40], [41]].

The combined effect of TiO_2_ and light on the antimicrobial activation was observed. Application of PL for 5 s significantly (*p* < 0.05) increased the biocidal effect of the TiO_2_ -PLA films and increase of TiO_2_ from P1 to P2 also enhanced the antimicrobial property (Fig. 3). It should be noted that TiO_2_ films without PL treatment also exhibited some biocidal effects (P1 and P2 in Fig. 3). This could be due to exposure of the films to fluorescent light while it was being prepared in the lab. Our preliminary study showed that the populations of *E. coli* on TiO_2_-PLA films were reduced by up to 2.0 log CFU/cm^2^ after exposure to fluorescent light in the lab for 48 h (data not shown), as compared to the films stored under dark conditions.

To differentiate the direct effect of PL treatments from results of activated TiO_2_ on microbial reduction, films were activated by PL prior to bacterial inoculation (pre-PL treatment). As displayed in Fig. 5, pre-PL treated film, without TiO_2_ (P0), possessed no antimicrobial properties, while films with TiO_2_ (P2) were reduced populations of *E. coli* and *Listeria* by 1.5–2.6 log CFU/cm^2^. These results suggest that the antimicrobial properties of TiO_2_ films activated by light could be preserved to some degree, although the antimicrobial effect was lower than that following immediate exposure to PL (Fig. 3). This may also explain why TiO_2_ films had some antimicrobial properties even without PL treatment (Fig. 3, Fig. 4), as TiO_2_ may have already been exposed to light, at some point, during manufacturing and storage.

Foods can be pasteurized before packaged or after packaged, the latter is called “In-package pasteurization”. To simulate the food packaging application for “In-package pasteurization”, the inoculated film surface was subjected to treatment with PL on the reverse side of the film (reverse treatment). That is, the PL needs to penetrate the film and activate the other side TiO_2_ particles that directly contact food. The reversed PL treatment reduced populations of both *E. coli* and *Listeria* by 1.5–1.8 log CFU/cm^2^ (Fig. 5). However, the reduction in microbial populations by the P2-RL film was less than that by PL treatment with P0-RL films. This is because TiO_2_ particles block light transmittance and absorb UV light. The images displayed in Fig. 2, and *T* in Table 1, indicate that light transparency decreased as TiO_2_ concentrations increased. In addition, titanium dioxide is an active ingredient in sunscreen, which helps protect human skin by blocking absorption of the sun's ultraviolet light. Therefore, reversed PL treatments reduced both the direct effect of PL and the effect of TiO_2_ on bacterial populations, which was dependent on the concentration of TiO_2_ in the film.

Food lipids are very susceptible to oxidation processes. UV light is one type of oxidation promoter. The exposure of food or packaged foods to UV light could lead to food lipid oxidation and degradation of food quality [42,43]. Therefore, it may be a significant challenge for PL treatments to achieve reasonable reductions of microbial populations without impacting food quality. On the other hand, we found that TiO_2_ films were able to store/preserve the antimicrobial energy following PL activation. Within 2 h of the 5 s PL treatment, TiO_2_ films still possessed antimicrobial activity and reduced bacterial populations by 2.5 log CFU/cm^2^ (Fig. 5). Therefore, PL pre-activated TiO_2_ film would have advantages for solid foods with ingredients which are sensitive to direct UV light treatment. It is also feasible for food processors to adopt this technology, since UV or pulsed light systems could be installed on packaging machines so that the packaging materials could be activated with PL, just prior to food being packaged. To the best of our knowledge, this is the first report of applying PL to pre-activate TiO_2_ particles within a PLA film, for the inactivation of *E. coli* or *L. innocua*.

Nevertheless, this was an initial feasibility study, limited to determining the antimicrobial properties of the packaging material, which did not address all the properties of the entire packaging system, such as physicochemical and mechanical properties of films. Future studies may evaluate physiochemical characterization of films and also assess the effectiveness of these films in real food samples, particularly those with fats. Furthermore, for commercial applications, an extrusion method should be employed to manufacture the films, hence, more related studies would be needed.

## 5 Conclusions

Antimicrobial PLA films, which incorporated TiO_2_ nanoparticles and were activated by pulsed light, were developed and their antimicrobial effects against *E. coli* and *Listeria* were determined based on three methods of pulsed light activation. In addition to direct PL activation, antimicrobial properties of TiO_2_-PLA films were also preserved, following activation by PL. Results from this feasibility study demonstrate that TiO_2_ -PLA materials have the potential to be used as a food-grade, biodegradable packaging material, and provide a novel approach to developing a new biodegradable and antimicrobial nanocomposite packaging material with potential application to food packaging.

## Data availability statement

Data will be made available on request.

## CRediT authorship contribution statement

**Tony Z. Jin:** Writing – review & editing, Supervision, Project administration, Methodology, Investigation, Funding acquisition, Conceptualization. **Xuetong Fan:** Writing – original draft, Methodology. **Sudarsan Mukhopadhyay:** Writing – original draft, Methodology.

## Declaration of competing interest

The authors declare that they have no known competing financial interests or personal relationships that could have appeared to influence the work reported in this paper.
